# Defining conversion therapy for esophageal squamous cell carcinoma

**DOI:** 10.1002/ags3.12623

**Published:** 2022-10-19

**Authors:** Satoru Matsuda, Takahiro Tsushima, Ken Kato, Chih‐Hung Hsu, Jang Ming Lee, Ian Yu‐Hong Wong, Hu‐Lin Christina Wang, Chang Hyun Kang, Xufeng Guo, Shun Yamamoto, Takayuki Tsuji, Hirofumi Kawakubo, Hiroya Takeuchi, Simon Law, Yuko Kitagawa

**Affiliations:** ^1^ Deepartment of Surgery Keio University School of Medicine Tokyo Japan; ^2^ Division of Gastrointestinal Oncology Shizuoka Cancer Center Shizuoka Japan; ^3^ Department of Head and Neck, Esophageal Medical Oncology National Cancer Center Hospital Tokyo Japan; ^4^ Division of Medical Oncology, Department of Oncology National Taiwan University Hospital Taipei Taiwan; ^5^ Department of Surgery National Taiwan University Hospital Taipei Taiwan; ^6^ Department of Surgery, School of Clinical Medicine The University of Hong Kong Hong Kong China; ^7^ Division of Traumatology Far Eastern Memorial Hospital Taipei Taiwan; ^8^ Department of Thoracic and Cardiovascular Surgery Seoul National University Hospital Seoul Korea; ^9^ Department of Thoracic Surgery, Shanghai Chest Hospital Shanghai Jiao Tong University Shanghai China; ^10^ Department of Surgery Hamamatsu University School of Medicine Hamamatsu Japan

**Keywords:** conversion surgery, conversion therapy, esophageal squamous cell carcinoma

## Abstract

A multimodality treatment conference with experts from across East Asia was held to establish a consensus for conversion therapy. An agreement was reached that conversion therapy was defined as surgery or chemoradiotherapy (CRT) aiming at cure after initial treatment for tumors that were initially unresectable due to adjacent organ invasion or distant metastasis.

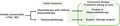

Systemic chemotherapy, with or without radiation, has been the standard treatment for locally advanced unresectable and/or metastatic esophageal squamous cell carcinoma (ESCC). There has been substantial progress in the multidisciplinary treatment of initially unresectable ESCC, which includes additional modalities such as immunotherapy.^1^, ^2^ The increased efficacy of multimodality treatment allows for the consideration of conversion therapy with curative intent for locally advanced unresectable and/or metastatic ESCC after initial treatment.

The concept of conversion therapy is well described in the treatment of unresectable gastric cancer. Conversion surgery has been defined as a surgical treatment aiming at an R0 resection after chemotherapy for tumors that were originally unresectable or marginally resectable for technical and/or oncological reasons. An international collaborative study, CONVO‐GC‐01, reviewed more than 1000 cases of stage IV gastric cancer patients who initially had unresectable disease but were able to undergo surgery with curative intent after systemic chemotherapy.^3^ Conversion surgery was adopted as one of the treatment options for Stage IV gastric cancer because of favorable long‐term outcomes, especially in patients with R0 resection. The AIO FLOT 3 trial evaluated whether neoadjuvant chemotherapy followed by surgical resection conferred a survival benefit to patients with gastroesophageal junction (GEJ) adenocarcinoma.^4^ The results demonstrated that 60% of patients with limited metastasis underwent surgery with favorable outcomes. Although conversion surgery is recognized as a treatment option in gastric and GEJ adenocarcinoma, it has not yet been established for ESCC.

ESCC is considered unresectable if there is adjacent organ invasion or distant metastasis. Furthermore, for ESCC conversion therapy with curative intent would be provided by surgical resection or definitive chemoradiotherapy. Therefore, the concept of conversion therapy needs to be discussed specifically for ESCC. For patients with ESCC with tumors that were initially unresectable due to adjacent organ invasion or distant metastasis, initial treatment involves either chemotherapy with/without immunotherapy or chemoradiotherapy (CRT). After initial treatment, in some patients surgery or CRT can be considered to cure the disease. We defined such local treatments aimed at curing the disease as conversion therapies for ESCC. When surgery is adopted as a conversion therapy following CRT, it is also called salvage surgery. A multimodality treatment conference with experts from across East Asia was held to establish a consensus for conversion therapy. An agreement was reached that conversion therapy was defined as surgery or CRT aiming at cure after initial treatment for tumors that were initially unresectable due to adjacent organ invasion or distant metastasis. The concept and definition of conversion therapy is described in Figure 1.

**FIGURE 1:**
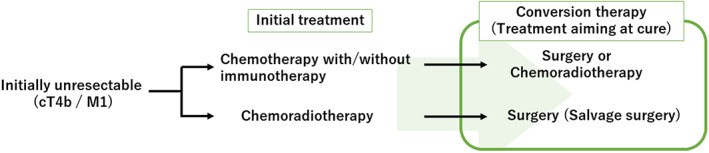
Definition of conversion therapy for esophageal squamous cell carcinoma

Given that the definition was established, we would expect to conduct a multicenter study to evaluate the safety and efficacy of conversion therapy for ESCC. A phase 3 study of tri‐modality combination therapy with induction docetaxel plus cisplatin and 5‐fluorouracil versus definitive chemoradiotherapy (JCOG1510) is ongoing for locally advanced unresectable ESCC with adjacent organ invasion.^5^

On the other hand, there is no consensus that conversion therapy is preferable for initially unresectable ESCC due to distant metastasis, and many clinical questions need to be addressed: (1) Does conversion therapy improve outcomes compared with continuous systemic treatment? (2) What treatment protocol is recommended as an initial treatment? (3) What are the indications for conversion therapy in unresectable ESCC due to distant metastasis? (4) Do outcomes for metastatectomy vary depending on the organ? (5) Does sequential systemic treatment improve survival after conversion therapy? (6) Is another local treatment, such as particle beam therapy, selected as a treatment modality for conversion therapy?

Furthermore, unlike gastric cancer, the surgical treatment for ESCC occasionally requires three‐field lymphadenectomy, and the incidence of postoperative complications is relatively high. Therefore, transthoracic esophagectomy accompanied by metastatectomy would be highly invasive. Additionally, the radiation field would need to be optimized based on the distribution of residual disease. To address all those questions, it would be valuable to develop an international consortium to conduct multicenter studies and to improve the treatment outcomes for advanced ESCC.

## DISCLOSURE

Conflict of Interest: Yuko Kitagawa reports grants and personal fees from Asahi Kasei Pharma Co., grants, personal fees, and other from Ono Pharmaceutical Co., Ltd., grants and personal fees from Otsuka Pharmaceutical Factory, Inc., grants and personal fees from Nippon Covidien Inc., grants, personal fees, and other from Taiho Pharmaceutical Co., Ltd, grants, personal fees, and other from Chugai Pharmaceutical Co., Ltd., grants, and personal fees from Kaken Pharmaceutical Co., Ltd., personal fees from AstraZeneca K.K., personal fees from Ethicon, Inc., personal fees from Olympus Co., personal fees from Shionogi & Co., Ltd., personal fees and other from Bristol‐Myers Squibb K.K., personal fees from MSD K.K., personal fees from Smith & Nephew KK, personal fees from Aska Pharmaceutical Co., Ltd., personal fees from Miyarisan Pharmaceutical Co. Ltd., personal fees from Toray Industries, Inc., personal fees from Daiichi Sankyo Company, Ltd., personal fees from Chugai Foundation for Innovative Drug Discovery Science, personal fees from Nippon Kayaku Co., Ltd., grants from Yakult Honsha Co. Ltd., grants from Otsuka Pharmaceutical Co., Ltd., grants from Tsumura & Co., grants from Sumitomo Pharma Co., Ltd., grants from EA Pharma Co., Ltd., grants from Eisai Co., Ltd., grants from Kyowa Kirin Co., Ltd., grants from Medicon Inc., grants from Takeda Pharmaceutical Co., Ltd., grants from Teijin Pharma Ltd., outside the submitted work; Ken Kato reports research grants from MSD; Ono Pharmaceutical Co.; Merck Serono; Bayer; Beigene; Oncologys Biopharma; Chugai Pharmaceutical Co.; and Shionogi. Satoru Matsuda, Hiroya Takeuchi, Simon Law, and Yuko Kitagawa are the editorial members of the *Annals of Gastroenterological Surgery*.
